# Histone modifications in head and neck squamous cell carcinoma

**DOI:** 10.3389/fonc.2024.1427725

**Published:** 2024-06-25

**Authors:** Wei Mao, Baoxin Wang, Ruofei Huang, Zhenfeng Sun, Minzhu Yan, Pin Dong

**Affiliations:** Department of Otolaryngology-Head and Neck Surgery, Shanghai General Hospital, Shanghai Jiaotong University School of Medicine, Shanghai, China

**Keywords:** head and neck squamous cell carcinoma (HNSCC), histone modification, methylation, acetylation, targeted therapies

## Abstract

Head and neck cancer is the main cause of cancer death worldwide, with squamous cell carcinoma (HNSCC) being the second most frequent subtype. HNSCC poses significant health threats due to its high incidence and poor prognosis, underscoring the urgent need for advanced research. Histone modifications play a crucial role in the regulation of gene expression and influencing various biological processes. In the context of HNSCC, aberrant histone modifications are increasingly recognized as critical contributors to its development and pathologic progression. This review demonstrates the molecular mechanisms, by which histone modifications such as acetylation, methylation, phosphorylation, and ubiquitination, impact the pathogenesis of HNSCC. The dysregulation of histone-modifying enzymes, including histone acetyltransferases (HATs), histone deacetylases (HDACs), and histone methyltransferases (HMTs), is discussed for its role in altering chromatin structure and gene expression in HNSCC. Moreover, we will explore the potential of targeting histone modifications as a therapeutic strategy, highlighting current preclinical and clinical studies that investigate histone deacetylase inhibitors (HDIs) and other epigenetic drugs, referring to the completed and ongoing clinical trials on those medications.

## 1 Introduction

**Figure 1:**
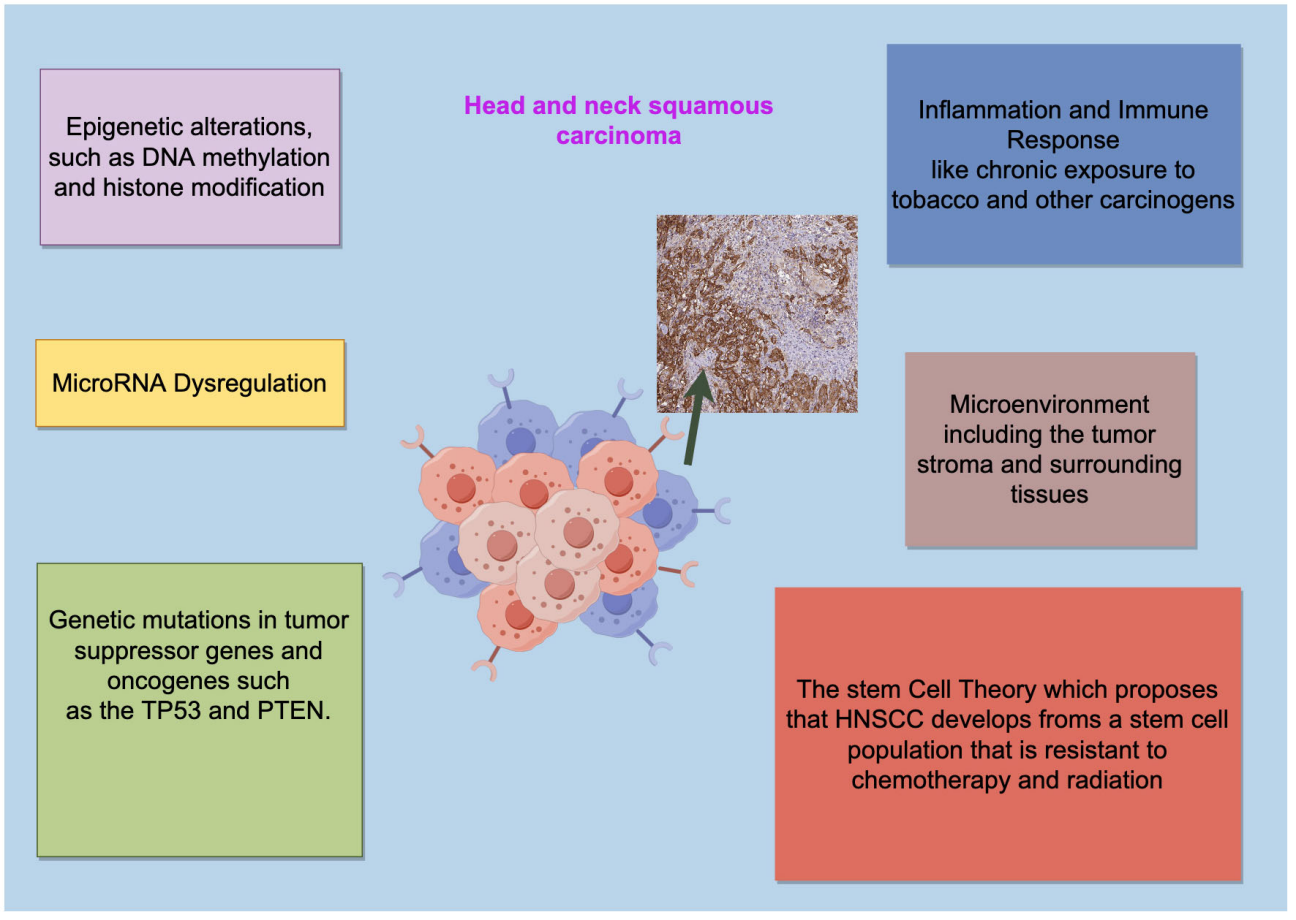
The pathogenesis of HNSCC is a multistep process. The pathogenesis of HNSCC is a complex multistep process influenced by multiple factors including epigenetic alterations, MicroRNA Dysregulation, genetic mutations in tumor suppressor genes and oncogenes and so on. Drawing using Figdraw tool.

**Figure 2:**
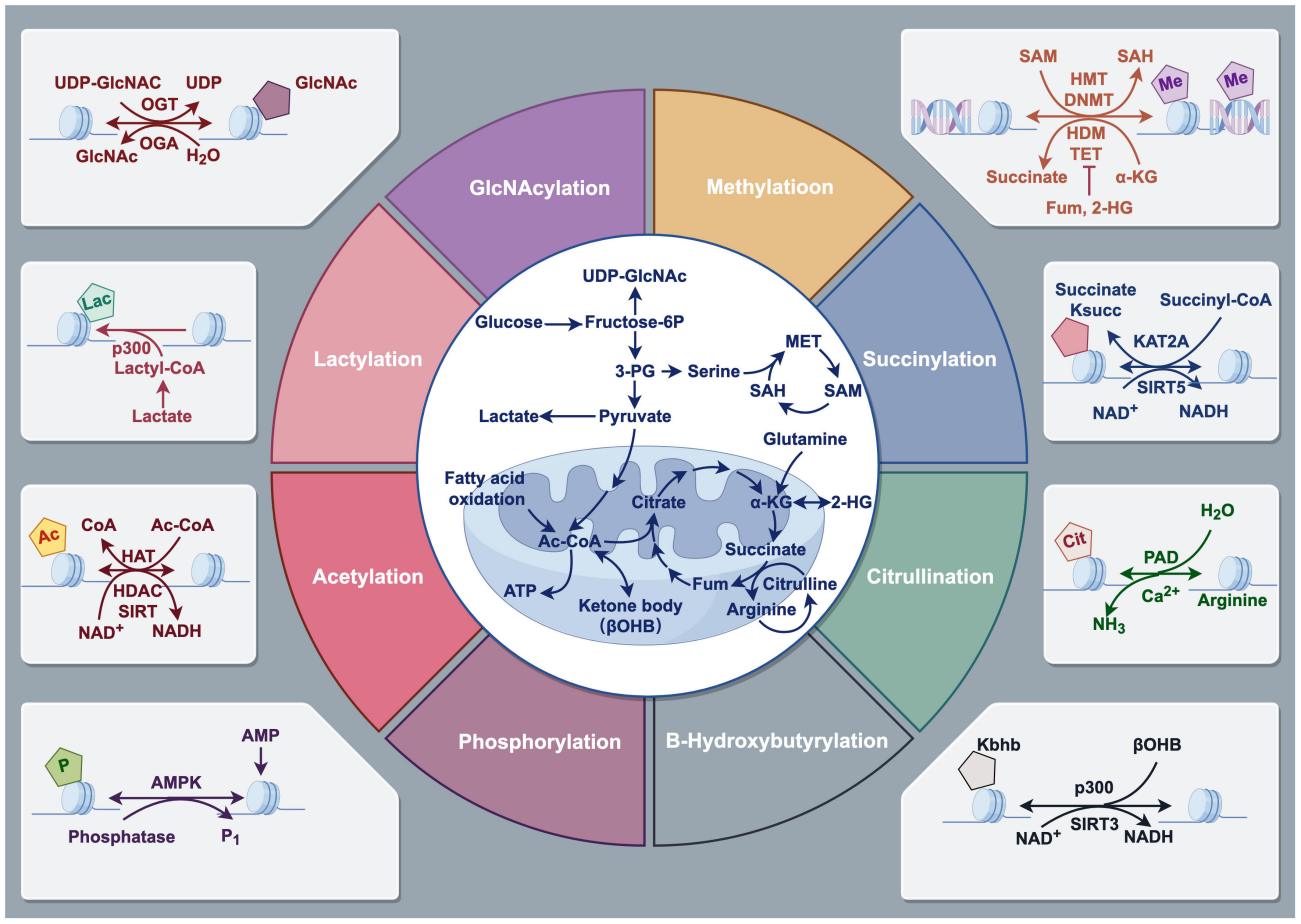
Types of Histone modifications. Histone modifications refer to the addition or removal of chemical groups, such as methyl, acetyl, phosphate, and ubiquitin, to specific amino acids on the histone protein tails. These modifications can alter the structure and function of chromatin, which is the complex of DNA and histones, and can either facilitate or inhibit gene expression. Histone modifications include methylation, acetylation, phosphorylation, ubiquitination, and many other forms. Drawing using Figdraw tool.

Exploring the pathogenesis of tumors to find effective treatments has always been a main theme of medical research. Head and neck squamous cell carcinoma (HNSCC) is one of the most common malignancies in the body, occurring mainly in the oral cavity, pharynx, and larynx. Globally, there are more than 600,000 new cases diagnosed each year, with a higher incidence in regions such as Southeast Asia and Central Europe. Characterized by high heterogeneity and mortality rate, about 60% of patients are already in the advanced stage when they seek medical help (1). It is well-known that genetic and epigenetic alterations are the main hallmarks of HNSCC tumorigenesis (**Figure 1**). Changes in the genetic material of the organism underlie all tumorigenesis, with gene mutations and chromosomal aberrations being the two primary aspects of genetic alteration. However, epigenetic changes are relatively complex, including DNA methylation, histone modifications, and the regulation of non-coding RNAs (2). Histone modification refers to the process of chemically modifying histones to alter their structure and function, they can activate or repress genes by changing the accessibility of DNA to transcription factors and other regulatory proteins, which is one of the most significant features of epigenetic changes. Histone modifications include methylation, acetylation, phosphorylation, ubiquitination, and many other forms (3) (**Figure 2**). Recent studies have highlighted the importance of specific histone modifications in HNSCC pathogenesis (4). This article provides a thorough overview of recent progress in comprehending the correlation between histone modification alterations and HNSCC. Certain modifications are deemed “classic” due to their early discovery, extensive examination, and frequent interactions, forming an intricate “histone code” capable of precisely regulating gene expression. By making these distinctions, our goal is to clarify the interactions among histone modifications, ultimately aiding in the discovery of more effective diagnostic markers and therapeutic targets for HNSCC.

## 2 Classic histone modifications in HNSCC

### 2.1 Histone methylation

**Figure 3:**
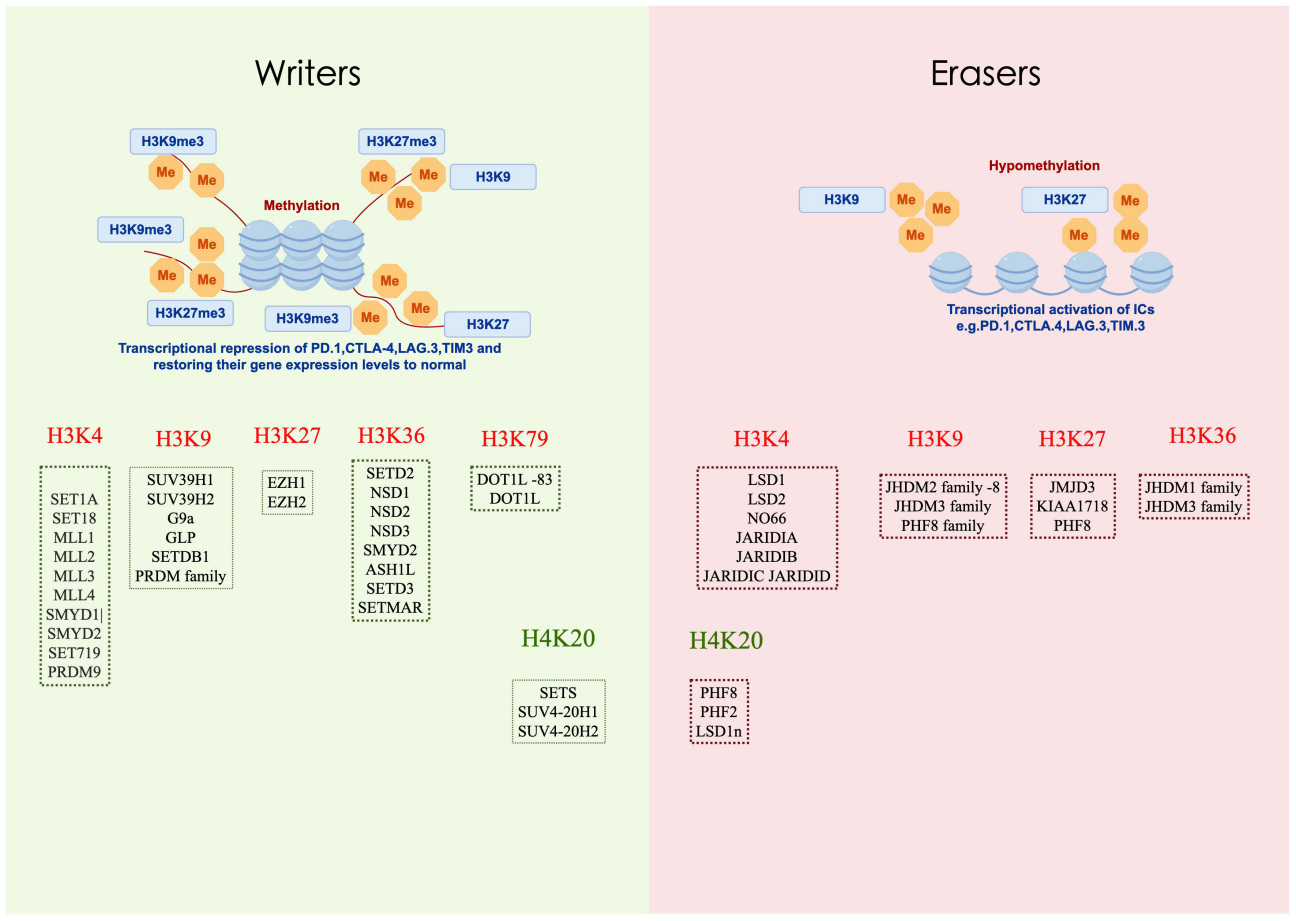
Writers and erasers in Histone Methylation. Histone methylation is a critical epigenetic modification involved in the regulation of gene expression. It involves the addition of one, two, or three methyl groups to specific lysine or arginine residues on histone tails. This modification is dynamically controlled by two classes of enzymes: “writers” and “erasers”. Drawing using Figdraw tool.

Histone methylation is a crucial post-translational modification in chromatin structure regulation, significantly impacting gene expression and cellular fate. This process predominantly involves the methylation of lysine and arginine residues on histones H3 and H4. Catalyzed by a series of specific enzymes, such as histone methyltransferases (HMTs) and histone demethylases (HDMs) (**Table 1**), histone methylation alters chromatin structure, thereby regulating gene activity. This modification is dynamic and reversible, for instance, H3K4me3 is typically associated with gene activation, whereas H3K27me3 correlates with gene silencing. These characteristics make histone methylation a pivotal mechanism in regulating gene expression and determining cellular fate (**Figure 3**).

**Table 1:** Common HMTs and HDMs.

Histone Methyltransferases (HMTs)				
Enzyme	Catalyzed Reaction	Target Lysine Residue	Function	
**SET1/MLL1**	H3K4 methylation	H3K4	Transcriptional activation	
**SET2**	H3K36 methylation	H3K36	Transcriptional elongation	
**EZH2**	H3K27 methylation	H3K27	Transcriptional repression, X chromosome inactivation	
**G9a**	H3K9 methylation	H3K9	Transcriptional repression, heterochromatin formation	
**SUV39H1**	H3K9 methylation	H3K9	Transcriptional repression, heterochromatin formation	
**SET7/9**	H3K4 methylation	H3K4	Transcriptional activation	
**DOT1L**	H3K79 methylation	H3K79	Transcriptional activation, DNA repair	
**PRDM2**	H3K9 methylation	H3K9	Transcriptional repression, DNA repair	
**PRDM14**	H3K27 methylation	H3K27	Transcriptional repression, pluripotency maintenance	
**NSD1**	H3K36 methylation	H3K36	Transcriptional activation, DNA repair	
**NSD2**	H3K36 methylation	H3K36	Transcriptional activation, DNA repair	
**NSD3**	H3K36 methylation	H3K36	Transcriptional activation, DNA repair	
**SET8**	H4K20 methylation	H4K20	Transcriptional repression, DNA repair	
**SMYD3**	H3K4 methylation	H3K4	Transcriptional activation, cell cycle regulation	

Histone Demethylases (HDMs)				
**Enzyme**	Catalyzed Reaction	Target Lysine Residue	Function	
**LSD1**	H3K4 demethylation	H3K4	Transcriptional repression, cell cycle regulation
**LSD2**	H3K4 demethylation	H3K4	Transcriptional repression, cell cycle regulation
**JARID1A**	H3K4 demethylation	H3K4	Transcriptional repression, cell cycle regulation
**JARID1B**	H3K4 demethylation	H3K4	Transcriptional repression, cell cycle regulation
**JARID1C**	H3K4 demethylation	H3K4	Transcriptional repression, cell cycle regulation
**JARID1D**	H3K4 demethylation	H3K4	Transcriptional repression, cell cycle regulation
**JMJDA**	H3K9 demethylation	H3K9	Transcriptional activation, cell cycle regulation
**JMJDB**	H3K9 demethylation	H3K9	Transcriptional activation, cell cycle regulation
**JMJDC**	H3K9 demethylation	H3K9	Transcriptional activation, cell cycle regulation
**UTX**	H3K27 demethylation	H3K27	Transcriptional activation, cell cycle regulation
**JMJD3**	H3K27 demethylation	H3K27	Transcriptional activation, cell cycle regulation
**FBXL11**	H3K36 demethylation	H3K36	Transcriptional repression, cell cycle regulation

In recent years, numerous studies have made remarkable achievements on the correlation between histone methylation and HNSCC. These investigations have revealed that antitumor immunity in HNSCC can be effectively induced by promoting DNA hypomethylation and derepression of retrotransposons through the induction of interferon response. Notably, the deactivation of histone H3K36 methyltransferase NSD1 leads to a depletion of H3K36 dimethylation (H3K36me2) and an elevation in H3K27 trimethylation (H3K27me3), which subsequently diminishes tumor immune infiltration (5). Through machine learning, another study screened 429 chromatin regulatory factors and discovered that histone methyltransferases enhance the stemness and migratory capabilities of tumor cells by upregulating SMYD3 and inducing H3K4 trimethylation (H3K4me3)-mediated HMGA2 expression (6). Current research suggests that approximately 20% of HNSCC cases exhibit reduced methylation of histone H3 at lysine 36 (H3K36me). This reduction is caused by mutations in the histone methyltransferase NSD1 or by lysine-to-methionine substitutions (H3K36M) in histone H3. Manipulation of the key epigenetic marker H3K27me3 can potentially play a supporting role in pharmacological treatment (7).

### 2.2 Histone acetylation imbalance

Histone acetylation is a process involves the addition of an acetyl group to the lysine residues in the histone proteins around which DNA is wound. This acetylation process is dynamic and catalyzed by histone acetyltransferases (HATs) (**Table 2**), specifically including B HAT and others primarily grouped into three families: GCN5/PCAF, CBP/p300, and the MYST family, which encompasses MOZ, MOF, TIP60, and HBO1. Among these, the MYST family stands as the largest subfamily of HATs, distinguished by a highly conserved MYST domain. This domain comprises a zinc finger and an acetyl-CoA binding motif. Additionally, HATs possess additional structures such as zinc fingers linked to plant homeodomains (seen in MORF and MOZ) and chromodomains (found in TIP60 and MOF). These structures contribute to the formation of protein complexes involved in both oncogenic and tumor suppressor activities. Another group of N-acetyltransferases (GNATs), represented by p300/CREB-binding protein (CBP), PCAF, HAT1, and GCN5, is associated with GCN5. Notably, GCN5 bears a bromodomain and catalyzes the acetylation of lysine residues on histones H2B, H3, and H4. The CBP/p300 subfamily comprises numerous subdomains capable of interacting with various proteins harboring disordered domains, such as p53 and NF-κB. The introduction of acetyl groups, which carry a negative charge, neutralizes the positive charge of lysine residues. This neutralization weakens the electrostatic attraction between histones and DNA. In contrast, histone deacetylases (HDACs) (**Table 2**) are capable of removing acetyl groups, thereby restoring the positive charge of histones, strengthening the binding between histones and DNA. This process results in a more condensed chromatin structure, ultimately inhibiting gene transcription. The balance between histone acetylation and deacetylation is crucial for the proper regulation of gene expression and is involved in numerous biological processes, including cell growth and development, DNA repair, and the response to environmental stimuli. Abnormalities in these processes can lead to diseases such as cancer and neurodegenerative disorders.

**Table 2:** HAT and HDAC.

Histone acetyltransferases(HAT)				
Family		Subfamily	Specific Enzymes	Chromosomal Location
**GNAT**			GCN5, PCAF, HAT1, HAT2	9q34, 16p13, 11q14, 6p22
		GNAT1	GCN5L2, HAT4	9q34, 12q24
		GNAT2	HAT3, HAT5	1p36, 14q24
**MYST**			MOZ, Ybf2, Sas2, Tip60, MORF	8p11, 14q32, 10q22, 5q33, 15q21
		MOZ1	MOZ1, MOZ2	8p11, 17q21
		MOZ2	MORF, HBO1	15q21, 12q13
**p300/CBP**			p300, CBP, Rtt109	22q13, 16p13, 5q31
**NCOA**			NCOA1, NCOA2, NCOA3	2p23, 8q12, 20q12
**TFIID**			TAF1, TAF2, TAF10	16q24, 19q13, 11q23
**Other**			NAT10, HAT7, HAT9, HAT10	2q31, 12q13, 11p15, 17q25

Histone deacetylases (HDAC)				
**Class**	HDAC Subfamily	Specific Name	Gene Symbol	Chromosomal Location
**I**	RPD3	Histone deacetylase 1	HDAC1	1p34.3
**I**	RPD3	Histone deacetylase 2	HDAC2	6q21
**I**	RPD3	Histone deacetylase 3	HDAC3	5q31.1
**I**	RPD3	Histone deacetylase 8	HDAC8	Xq13.1
**IIa**	HDAC4	Histone deacetylase 4	HDAC4	2q37.3
**IIa**	HDAC5	Histone deacetylase 5	HDAC5	17q21
**IIa**	HDAC7	Histone deacetylase 7	HDAC7	15q24.2
**IIa**	HDAC9	Histone deacetylase 9	HDAC9	7p21.1
**IIb**	HDAC6	Histone deacetylase 6	HDAC6	Xp11.23
**IIb**	HDAC10	Histone deacetylase 10	HDAC10	22q13.31
**III**	Sirtuin	Sirtuin 1	SIRT1	10q21.3
**III**	Sirtuin	Sirtuin 2	SIRT2	19q13.2
**III**	Sirtuin	Sirtuin 3	SIRT3	11p15.5
**III**	Sirtuin	Sirtuin 4	SIRT4	12q24.23
**III**	Sirtuin	Sirtuin 5	SIRT5	6p23
**III**	Sirtuin	Sirtuin 6	SIRT6	19p13.3
**III**	Sirtuin	Sirtuin 7	SIRT7	17q25.3
**IV**	HDAC11	Histone deacetylase 11	HDAC11	3p25.3

Most tumor cells exhibit lower levels of histone acetylation and maintain an acidic intracellular environment, with HNSCC serving as a prime example of this phenomenon (8). Through whole-exome sequencing of human HNSCC tumors (n=235), studies have confirmed that mutations in histone acetyltransferases CREBBP, EP300, or CASP8 are associated with poor prognosis after radiotherapy for HNSCC. This effect is achieved by inhibiting HAT function, thereby suppressing homologous recombination and increasing DNA damage (9). On the other hand, experimental evidence currently suggests that HDAC1 and HDAC2 can bind to ΔNp63α, a prognostic marker for HNSCC, forming an active transcriptional repressor complex that promotes tumor progression (10). Mounting evidence from similar studies converges to suggest that modulating histone dysregulation has emerged as a promising therapeutic strategy for HNSCC treatment. Several drugs have been shown to exert their therapeutic effects by targeting this pathway. Cetuximab (11), for instance, a monoclonal antibody that targets the epidermal growth factor receptor (EGFR), has been studied extensively in the treatment of HNSCC. A crucial aspect of Cetuximab’s pharmacological mechanism involves its ability to suppress tumor cell proliferation by modulating the activity of Acetyl-CoA Carboxylase (ACC). ACC is a metabolic enzyme, and its activity dysregulation is closely linked to the progression of various solid tumors. numerous studies indicate a possible correlation between alterations in acetylation status within tumor cells and Cetuximab therapy resistance. Modulating the activity of HDACs and HATs may potentially overcome Cetuximab resistance, thereby improving therapeutic efficacy (12). Similar compounds such as ACY-241 (13), and JQ1 (14), have also been found to exert their therapeutic effects by targeting this pathway, offering a new avenue for treatment exploration.

### 2.3 Histone phosphorylation

Histone phosphorylation is a post-translational modification (PTM) that plays a crucial role in regulating chromatin structure and gene expression. It involves the addition of a phosphate group to specific amino acid residues (usually serine, threonine, or tyrosine) on histone proteins. This process is catalyzed by protein kinases, typically utilizing ATP as the source of phosphate groups. Phosphorylation modification may reduce the binding affinity of histones to DNA, thereby facilitating DNA unwinding and the binding of transcription factors. This, in turn, stimulates gene transcription and expression. Research reveals that histone phosphorylation levels are abnormally elevated in cancer cells across multiple tumor types, a phenomenon closely correlated with cellular proliferation, metastasis, and invasion capabilities. For instance, studies have shown that histone H3 phosphorylation at serine 10 (H3S10ph) is significantly increased in various cancers, including breast cancer and colorectal cancer, and is associated with poor prognosis. Current research has revealed that in HNSCC, not only do histones themselves, such as H1 (15) and H3, H4 (16), undergo modifications, but specific amino acid residues like H3Ser10 (17) are also phosphorylated to regulate tumor progression. In HNSCC, abnormal histone phosphorylation activates various signaling pathways, including PI3K/Akt, MAPK, and JNK. The activation of these oncogenic signaling pathways and the inactivation of tumor suppressor pathways accelerate the progression of HNSCC. Tumor therapeutic drugs are developed based on these findings. For instance, PI-828 and PI-103 can block PI3K, affecting downstream signaling molecules such as Akt and mTOR, thereby inducing apoptosis in HNSCC cells, altering cell cycle regulation, and reducing invasiveness (18). Additionally, cetuximab has been reported to activate the epidermal growth factor receptor in HNSCC cell lines, regulating tyrosine 1173 hyperphosphorylation (19) and contributing to therapeutic effects.

### 2.4 Histone ubiquitylation

Ubiquitin, a highly conserved small protein, conjugates with protein substrates through a stepwise enzymatic cascade involving E1 ubiquitin-activating enzyme, E2 ubiquitin-conjugating enzyme, and E3 ubiquitin ligase. This process plays a crucial role in various cellular processes, including protein degradation, cell signaling, DNA repair, and cell cycle regulation. Defined as the covalent attachment of ubiquitin molecules to target proteins, ubiquitination stands as one of the most prevalent post-translational modifications within the proteome. Structurally, ubiquitin linkage can be classified as three patterns: monoubiquitination, polyubiquitination, and branched ubiquitination. Notably, each type of ubiquitination, alongside lysine ubiquitin linkage occurrences, triggers unique biological responses.

In HNSCC, the involvement of ubiquitin is particularly crucial as it impacts the stability and functionality of oncogenic proteins and tumor suppressor genes, ultimately influencing tumor development and progression. Several genome-wide studies have revealed common mutations/deletions in the CYLD and TRAF3 genes in HNSCC. CYLD, a crucial gene in clear K63 polyubiquitin and M1 linear ubiquitin chains, inhibits NFκB signaling activation at multiple distinct steps (20). Furthermore, TRAF3 serves as a negative regulator of both canonical and non-canonical NFκB pathways, and its deletion can enhance tumor stemness (21). Mutation analysis further indicates that SCFFBXW7, the most frequently mutated gene in HPV- HNSCC, promotes the stabilization of NFκB protein p100 by reducing its degradation through the influence of E3 ligase. This may potentially increase the inactive precursor induced by stimulation, thereby enhancing NFκB activity (22). In a study adopted customized siRNA and cDNA libraries for deubiquitinating enzymes, it was discovered that USP7 serves as the bona fide TAZ deubiquitinase in HNSCC. USP7 promotes cell proliferation, migration, *in vitro* invasion, and tumor growth by stabilizing TAZ. USP7 interacts with TAZ to deubiquitinate and stabilize it by selectively removing its K48-linked ubiquitin chains. Inhibition of USP7 significantly suppresses tumor growth in HNSCC xenografts and PDX models (23). Furthermore, ubiquitin-targeted therapies show a promising future in HNSCC. The development of isoform-specific E3 ligase inhibitors or DUB inhibitors could potentially minimize off-target effects and enhance therapeutic selectivity (24). Additionally, identifying novel ubiquitin-binding domains (UBDs) and elucidating their roles in HNSCC may contribute to the discovery of new drug targets.

## 3 Novel histone modifications in HNSCC

### 3.1 Histone succinylation

Histone succinylation is a recently discovered PTM that plays a crucial role in regulating chromatin structure, gene expression, and cellular processes. Succinylation is the covalent attachment of a succinyl group (CH3CH2CO-) to a specific amino acid side chain on a histone protein. This modification is carried out by enzymes called succinyltransferases, which transfer the succinyl group from succinyl-CoA to the target histone. Succinylation can occur on multiple histone proteins, including H2A, H3, and H4. Histone succinylation can impact the structure and function of chromatin. This modification may lead to chromatin relaxation, promoting transcriptional activity of genes, or regulating gene expression through other mechanisms. Research indicates that histone succinylation plays a significant role in the development and progression of HNSCC. The succinylation levels of histones H3K9, H3K14, and H3K56 are notably elevated in HNSCC tissues, correlating with tumor staging and prognosis (25). Another study suggests that histone H3K122 succinylation promotes HNSCC cell proliferation and invasion by regulating the NOTCH1 signaling pathway (26).

Given the significant role of histone succinylation in HNSCC, targeting this epigenetic modification has emerged as a new therapeutic strategy for HNSCC treatment. EPZ-6438, also known as Tazemetostat, is a small molecule drug belonging to the class of histone methyltransferase (EZH2) inhibitors. Its primary function is to inhibit the activity of the EZH2 enzyme. It has been found to target histone H3K27 succinylation (5), thereby inhibiting the proliferation and invasion of HNSCC cells and inducing apoptosis. Another study revealed that the histone deacetylase inhibitor SAHA (27) exerts its therapeutic effect by inhibiting the succinylation of histones H3K9 and H3K56 (28). These studies suggest that targeting histone succinylation represents a promising treatment strategy for HNSCC, which can be used alone or in combination with traditional chemotherapy drugs to enhance therapeutic outcomes and reduce side effects.

### 3.2 Histone lactylation

Histone lactylation involves the attachment of a lactyl group (composed of a hydroxyl and a carboxyl group) to the lysine residues of histone proteins. This process is catalyzed by enzymes known as histone lactylases, which belong to the Sirtuin protein family. Utilizing NAD+ as a cofactor, these enzymes facilitate the transfer of a lactyl group from β-alanine to the ϵ-amino group of lysine residues on histone proteins. Notably, this reaction is reversible, implying that the lactyl group can be detached by an enzyme designated as histone lactylase-removing enzyme (LLRE). In HNSCC, research on histone lactylation mainly focuses on three aspects: transcription regulation, DNA repair mechanisms, and cell cycle control. Studies have indicated the abnormal expression of various enzymes associated with histone lactylation in HNSCC. Specifically, the expression of histone acetyltransferase P300/CBP is significantly upregulated in HNSCC, whereas the expression of histone deacetylases HDAC1 and HDAC2 is notably downregulated (29). Furthermore, the expression levels of histone acetyltransferase P300/CBP and HDAC1 in HNSCC correlate with the malignancy and prognosis of the tumor (30).

Given the pivotal role of lactate in tumorigenesis and progression, research targeting histone lactylation is becoming a hot topic. GPR81, standing for G protein-coupled receptor 81 and also known as lactate receptor 1, is a G protein-coupled receptor reported to be widely expressed in various solid tumors, including HNSCC. GPR81 is intricately linked to tumor growth and metastasis. Silencing GPR81 has been shown to exert effective therapeutic effects (31).

### 3.3 Histone glycosylation

Histone glycosylation involves the addition of a carbohydrate moiety, specifically a sugar molecule, to the histone proteins. This modification is catalyzed by a family of enzymes called histone O-glycosyltransferases, which are responsible for adding a specific sugar molecule, such as N-acetylglucosamine (O-GlcNAc), to the histone proteins. Histone glycosylation occurs at specific serine and threonine residues on the histone proteins, which are recognized by the glycosyltransferases. Histone glycosylation modifications can be primarily categorized into two types: O-GlcNAzylation and ADP-ribosylation. O-GlcNAzylation refers to the process of attaching an N-acetylglucosamine group to the serine or threonine residues of a protein, whereas ADP-ribosylation involves the addition of an ADP-ribose group to the glutamic acid or aspartic acid residues of a protein. Studies have found that O-GlcNAc transferase (OGT)-mediated glycosylation modification at the H2BSer112 site of histone is significantly upregulated in various solid tumors such as HNSCC. Knockdown of OGT can inhibit tumor cell proliferation and induce apoptosis (32). Additionally, Lin et al. (33) found that the glycosyltransferase GALNT2 increased in HNSCC through immunohistochemistry, this increasement can regulates tumor growth via the EGFR/AKT signaling axis. OSMI-1, a cell-permeable O-GlcNAc transferase (OGT) inhibitor, inhibit protein O-GlcNAcylation without qualitatively altering N- or O-linked glycans on the cell surface. In a study targeting esophageal squamous cell carcinoma, it was determined that OSMI-1 can exert a therapeutic effect by regulating PD-1 in tumor cells (34), the above results suggesting that targeting OGT-mediated histone O-GlcNAcylation modification may be a promising anti-HNSCC strategy.

### 3.4 Histone β-hydroxybutyrylation

Histone β-hydroxybutyrylation (Kbhb) is the addition of a β-hydroxybutyryl group to the lysine residues of histone proteins. This modification is catalyzed by a family of enzymes called histone β-hydroxybutyryltransferases (H3BTs), which use β-hydroxybutyrate as a cosubstrate. This modification usually occurs on the acetyl groups of histones H3 and H4, leading to structural changes in the histones, which subsequently affect the state of chromatin and gene expression. Current research indicates a close relationship between Kbhb and metabolic pathways. The elevation of histone H3K9bhb levels during bodily hunger is associated with the upregulation of genes in hunger-responsive metabolic pathways, and p300-catalyzed histone Kbhb can directly activate transcription (35). Although nowadays research on Kbhb in the field of squamous cell carcinoma is limited, certain enzymes or genes in this pathway have been shown to impact HNSCC. For instance, HMGCS2, which encodes 3-hydroxy-3-methylglutaryl-CoA synthase (HMGCR), is a crucial enzyme in cholesterol biosynthesis and ketogenesis. HMGCS2 can reduce beta-hydroxybutyrate (β-OHB) levels, thereby decreasing histone kbhb modifications, highlighting a tight relationship between the two. A study analyzing HMGCS2 levels in patients with esophageal squamous cell carcinoma (ESCC) revealed significant downregulation in primary ESCC, correlating strongly with patient prognosis (36). Additionally, metformin, a well-known drug, has been proven to increase Kbhb levels in the body. It has been demonstrated to downregulate gene expression profiles associated with cancer stemness and epithelial-mesenchymal transition while increasing the expression of squamous differentiation genes, thus preventing the development of HNSCC (37). In summary, there is a certain correlation between Kbhb and HNSCC, which may hold significant importance in gene expression regulation, metabolic reprogramming, and potential disease treatments.

### 3.5 Other novel histone modifications in HNSCC

Several recent studies have shown that there are also multiple types of histone modifications that are associated with the development and treatment of HNSCC. Histone crotonylation, for instance, is the addition of a crotonyl group (-CH=CH-COO-) to the lysine residues of histone proteins. This modification is catalyzed by a family of enzymes called histone crotonyltransferases (KAT5/8), which use crotonyl-CoA as a cosubstrate. Histone crotonylation can activate or repress the transcription of specific genes in HNSCC by altering chromatin structure, thereby influencing disease progression (38). Through sequencing and enrichment analysis, it has been found that histone malonylation of HSP90AB1 mediates the onset and progression of oral squamous cell carcinoma (39). With understanding of histone modifications deepens, researchers have reported novel modification patterns like histone SUMOylation. SUMO (Small Ubiquitin-like MOdifier) is a small protein that is similar in structure to ubiquitin, a protein that plays a key role in protein degradation which is also known as sentrin or SMT3. Unlike ubiquitination, SUMOylation does not promote the degradation of its target proteins. Instead, SUMOylation has been shown to regulate various aspects such as protein stability, subcellular localization, protein interactions, and transcriptional regulation (40). Currently, researchers have intervened in the development of HNSCC by applying SUMO-1 inhibitors (41). As these studies progressed, they have improved our understanding of the mechanisms of HNSCC occurrence.

## 4 Summary and prospects

In summary, the role of histone modifications in the pathogenesis of HNSCC is increasingly recognized as a critical factor in tumor development and progression. Key findings from recent studies underscore the significant impact of histone acetylation, methylation, and other modifications on gene expression and cellular behavior in HNSCC. Recent advancements in molecular biology, genomics, and proteomics have broaden our insights on the mechanisms underlying HNSCC development. For instance, aberrant histone deacetylation and methylation have been linked to the repression of tumor suppressor genes and activation of oncogenes, contributing to the malignant phenotype. The significance of these findings lies in the potential for histone modifications to serve as therapeutic targets (**Table 3**), metaphorically operating as a covert puppeteer manipulating various tumorigenic events. This review delves into the profound impact of histone modifications in HNSCC and summarizes the latest research on targeting these modifications as a therapeutic strategy.

**Table 3:** Histone modifying factors in HNSCC.

Histone Modifying Enzyme	Type of Modification	Role in HNSCC	Therapeutic Agents	Clinical Trial Status
HDAC1	Deacetylation	Promotes oncogene expression and represses tumor suppressor genes	Vorinostat, Romidepsin	Phase II (NCT01928576)
HDAC2	Deacetylation	Similar to HDAC1, involved in transcriptional repression	Panobinostat, Belinostat	Phase I/II (NCT01396263)
HDAC3	Deacetylation	Linked to DNA damage response and inflammation	Entinostat, Valproic acid	Investigational
EZH2	Methylation (H3K27me3)	Silences tumor suppressor genes; associated with poor prognosis	Tazemetostat, EPZ-6438	Phase II (NCT03854474)
SUV39H1	Methylation (H3K9me3)	Involved in heterochromatin formation and gene silencing	Chaetocin	Preclinical
DOT1L	Methylation (H3K79me)	Promotes transcriptional activation; linked to cell cycle regulation	Pinometostat	Early-phase trials
PRMT5	Methylation	Regulates RNA splicing, growth, and migration	JNJ-64619178	Phase I (NCT03573310)
CBP/p300	Acetylation (H3 and H4)	Acts as a transcriptional coactivator; mutation linked to oncogenesis	A-485, CCS1477	Investigational
Tip60	Acetylation (H4K16)	Involved in DNA repair and apoptosis	TH1834	Preclinical
LSD1	Demethylation (H3K4me1/2)	Promotes proliferation and metastasis	ORY-1001, Seclidemstat	Phase II (NCT03155620)
JARID1B/KDM5B	Demethylation (H3K4me3)	Implicated in tumor growth and drug resistance	CPI-455, GSK-J4	Preclinical

**Figure 4:**
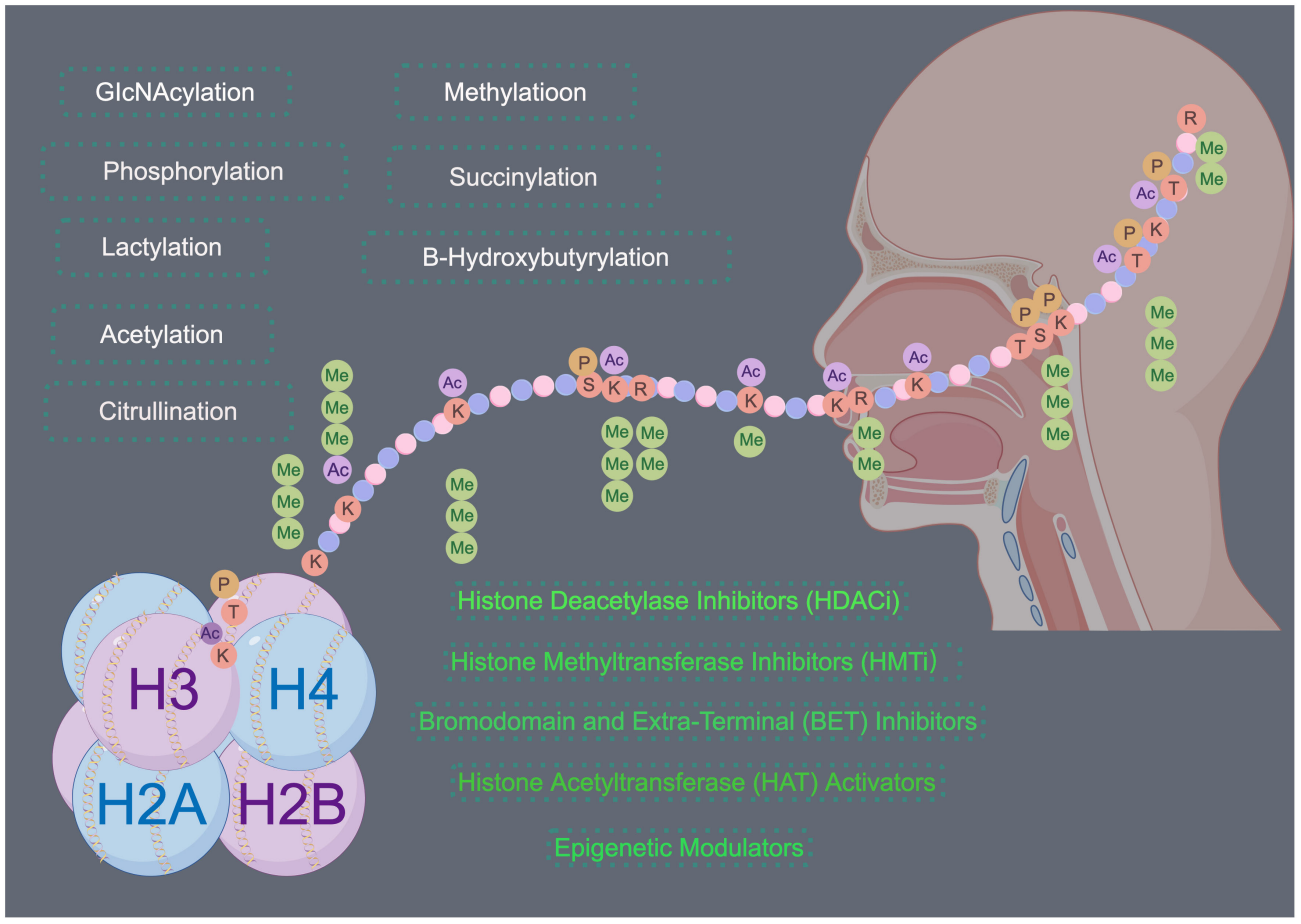
Histone modifications in HNSCC. Histone modifications play a significant role in the development and progression of HNSCC. Targeting these modifications through inhibition of histone-modifying enzymes or direct targeting of histone modifications offers a promising therapeutic strategy for the treatment of HNSCC. Drawing using Figdraw tool.

In HNSCC the equilibrium of histone modifications becomes disrupted, ultimately leading to altered gene expression patterns that drive tumorigenesis and disease progression (**Figure 4**). The primary results of this disruption can be categorized into three primary aspects: the transcriptional dysregulation of oncogenes and tumor suppressor genes mediated by abnormal histone modifications, the modulation of the stem-like properties of HNSCC tumor cells, and the perturbation of the tumor microenvironment. Despite the expanding numbers of research in this field, numerous gaps persist, primarily due to the intricate nature of histone post-translational modifications (PTMs). Recently, several histone modifications have emerged as potent prognostic indicators in HNSCC, offering valuable insights into patient survival and disease trajectory. For example, Trimethylation of histone H3 lysine 27 (H3K27me3) is associated with gene repression and is often found in the tumor suppressor gene loci. High levels of H3K27me3 have been correlated with poor prognosis and decreased overall survival in HNSCC patients. Ubiquitination of histone H2B lysine 5 (H2BK5ub) has been shown to play a critical role in immune evasion and tumor progression in HNSCC. High levels of H2BK5ub have been linked to increased tumor immune infiltration, which is associated with improved prognosis and better response to immunotherapy. Concurrently, research efforts focused on the development of drugs targeting specific histone-modifying enzymes are progressing, thereby carving new therapeutic avenues for HNSCC. Furthermore, the invention of transferase inhibitors, notably DNA methyltransferase inhibitors (DNMTis) and histone deacetylase inhibitors (HDACis), has enriched the therapeutic methods for HNSCC, by effectively targeting abnormal histone modifications and impeding tumor proliferation and metastasis.

Future research should focus on several key areas. First, there is a need for comprehensive studies to map the global landscape of histone modifications in HNSCC. This includes identifying specific histone marks associated with different stages of tumorigenesis and their prognostic significance. Second, understanding the interplay between histone modifications and other epigenetic mechanisms, such as DNA methylation and non-coding RNAs, could provide deeper insights into the complex regulatory networks driving HNSCC. Moreover, clinical trials are necessary to evaluate the safety and efficacy of epigenetic therapies targeting histone modifications in HNSCC patients. Such trials should explore combination therapies, integrating epigenetic drugs with conventional treatments like chemotherapy, radiotherapy, and immunotherapy, to assess potential synergistic effects. We are optimistic that a deeper understanding of abnormal histone modifications in HNSCC will allow us to overcome current therapeutic limitations, and provide patients with more precise and personalized treatment options. This will vastly improve patient prognosis, giving renewed hope and energy to those fighting the disease. We eagerly await further research progress in this field.

## Author contributions

PD: Resources, Writing – review & editing. WM: Conceptualization, Writing – original draft, Writing – review & editing. BW: Resources, Writing – review & editing. RH: Resources, Writing – review & editing. ZS: Resources, Writing – review & editing. MY: Resources, Writing – review & editing.
